# CHEMOBRAIN: Cognitive Deficits and Quality of Life in Chemotherapy Patients—Preliminary Assessment and Proposal for an Early Intervention Model

**DOI:** 10.3390/cancers18010066

**Published:** 2025-12-24

**Authors:** Erika Cavalletto, Pamela Iannizzi, Eleonora Bergo, Daniela Grosso, Giorgia Gasparotto, Alessandra Feltrin, Nicola Galtarossa, Matteo Bernardi

**Affiliations:** 1 Healthcare Professions, Veneto Institute of Oncology IOV-IRCCS, 35128 Padua, Italy; erika.cavalletto@iov.veneto.it (E.C.); daniela.grosso@iov.veneto.it (D.G.); nicola.galtarossa@iov.veneto.it (N.G.); matteo.bernardi@iov.veneto.it (M.B.); 2 Hospital Psychology, Veneto Institute of Oncology IOV-IRCCS, 35128 Padua, Italy; pamela.iannizzi@iov.veneto.it (P.I.); giorgia.gasparotto@studenti.iusve.it (G.G.); alessandra.feltrin@iov.veneto.it (A.F.)

**Keywords:** chemotherapy, cancer-related cognitive impairment (CRCI), quality of life, FACT-Cog, chemobrain, psychoeducation

## Abstract

**Background/Objectives:** Chemotherapy can negatively affect cognitive functioning through direct and indirect mechanisms, with 15–50% of patients experiencing a decline that impacts quality of life. While neurocognitive tests are the main assessment tool, self-reported measures provide valuable additional insights. The aim of this study was to evaluate the cognitive decline of patients during the early stages of chemotherapy treatment and its impact on their quality of life, as well as to outline future perspectives for an early intervention model. **Materials and Methods:** This prospective longitudinal study was conducted on 40 patients aged 18 to 64 years. Data were collected using the FACT-Cog v.3 questionnaire, administered at the beginning of the first chemotherapy cycle (T0), and then again after approximately 3–4 weeks (T1) and 6–8 weeks (T2). **Results:** The data show a progressive decline in perceived cognitive abilities (PCI: 72.10 → 64.43; PCA: 25.70 → 20.90) and overall quality of life (9.73 → 8.75) from T0 to T2. A significant positive correlation was found between perceived cognitive abilities and quality of life, supporting the link between cognitive decline and quality of life. Questionnaire variables reliably predicted changes in quality of life two months after chemotherapy [F(4, 35) = 3.91, *p* = 0.01]. **Conclusions:** The findings show a decline in quality of life as chemotherapy progresses. However, this decline can be predicted from the second month of treatment using the FACT-Cog v.3 questionnaire. The findings further highlight even more the value of integrating early empowerment, rehabilitative and psychoeducational programs to mitigate cognitive decline and improve quality of life.

## Simple Summary

Chemotherapy can cause cognitive decline, known as cancer-related cognitive impairment (CRCI), affecting memory, attention, processing speed, and executive functions. This research was conducted to better understand how cognitive difficulties arise during the first weeks of chemotherapy and how they impact daily life. Forty patients aged 18–64 completed a questionnaire (FACT-Cog v.3) at the start of treatment and during the following two months to report their own experiences with memory, attention, and mental clarity. We observed a gradual decline in how patients perceived their cognitive functioning, which was closely linked to a worsening quality of life. Importantly, these early self-reported changes were able to predicted subsequent declines. This suggests that simple, early assessments could help identify patients who might benefit from timely support. Our findings encourage the development of early intervention programs to maintain cognitive well-being and improve the overall experience of patients undergoing chemotherapy.

## 1. Introduction

Chemotherapy is a well-established treatment for many types of cancer and is one of the main strategies adopted in oncology. It is often used alongside local treatments such as surgery and radiotherapy [1,2,3]. Chemotherapeutic agents inhibit cellular proliferation by interfering with the underlying mechanisms of this process [4,5,6]. Side effects such as alopecia, anemia, immunosuppression and vomiting can sometimes be perceived by patients as more distressing, and can have a profound impact on quality of life during and after treatment. In response to these challenges, a major evolution has occurred in the therapeutic landscape, with the introduction of chemotherapeutic regimens that are both more effective and less toxic than those historically employed.

Less visible, yet equally impactful, are the cognitive consequences of chemotherapy. These have been extensively documented in studies reporting cognitive decline in 15% to 50% of patients, with incidence rates reaching up to 75% [1]. Patients undergoing treatment often report a general sense of mental fogginess, which is defined as cancer-related cognitive impairment (CRCI)—commonly referred to as ‘chemobrain’ or ‘chemofog’—and encompasses symptoms affecting cognitive domains such as memory, language, attention, processing speed, executive functions, and visuospatial abilities [7,8]. CRCI can significantly impact the lives of affected individuals, limiting their daily, social, professional and educational activities [9]. While the exact mechanisms by which chemotherapy causes brain damage are unclear, neuroimaging studies have consistently revealed structural and functional changes in various partes of the brain of patients receiving systemic chemotherapy [10,11,12,13].

Understanding the neurobiological and psychological mechanisms of CRCI is, therefore, crucial for developing effective interventions to mitigate its effects [14].

Cognitive decline can be assessed using objective methods, such as structured neurocognitive tests, or subjective approaches, such as self-report questionnaires [15]. While the ICCTF (International Cognition and Cancer Task Force) identifies neurocognitive tests as the gold standard for evaluating cognitive functioning in cancer patients [16,17,18], several studies have indicated that these tools fail to capture patients’ subjective experiences or their impact on daily life [17,18,19,20,21,22].

The Veneto Institute of Oncology has a neurocognitive clinic is available, which uses standardized tools to assess cognitive disorders and cognitive rehabilitation, including cognitive training and psychoeducational programs.

We conducted a study to enable early assessment of these CRCIs in patients undergoing the initial cycles of chemotherapy through self-evaluation questionnaires.

In this study we present the results of the preliminary assessment and we propose an early cognitive intervention model.

## 2. Materials and Methods

This observational, non-profit study employed a prospective longitudinal design. Starting with the selection of patients exposed to a risk factor (chemotherapy), it investigated the onset of perceived cognitive decline and its impact on quality of life.

Participants were recruited from among patients attending the Day Hospital of the Veneto Institute of Oncology in Padua.

The inclusion criteria were age ≥ 18 years, diagnosis of non-CNS cancer and treatment with intravenous chemotherapy. The exclusion criteria were as follows: age > 65 years (to exclude the effect of biological aging), diagnosis of a primary brain tumor, presence of brain metastases, confirmed neurodegenerative or cognitive disorders and prior cycles of chemotherapy.

The study design entailed completing a questionnaire at three time points: at the first chemotherapy session (T0); after 3–4 weeks (T1), depending on therapy protocols; and after 6–8 weeks (T2).

Data collection took place during chemotherapy sessions. Each participant was given a brief written summary of the study, including its rationale and the methods used to collect data, as well as a consent form for data processing and a form to collect demographic information (sex, age and diagnosis).

Cognitive decline and quality of life were assessed using the Italian version of the Functional Assessment of Cancer Therapy—Cognitive Function v.3 (FACT-Cog) questionnaire [23,24]. This tool is divided into domains or subscales:

- COG-PCI (Perceived Cognitive Impairment), evaluating cognitive symptoms experienced by the participant;
- COG-O (Others), focusing on comments from others on the participant’s cognitive status;
- COG-PCA (Perceived Cognitive Abilities), measuring cognitive abilities perceived by the participant;
- COG-Q (Quality of Life), exploring the impact of cognitive difficulties on overall quality of life through four items.

In each subscale, higher scores indicate a better self-reported quality of life.

The study was approved by the local ethics committee and conducted in accordance with the ethical standards set out in the Declaration of Helsinki.

## 3. Statistical Analysis

Data were analyzed using MS Excel and Stata MP (Stata 14). Descriptive statistics were first applied to characterize the sample by calculating the absolute and relative frequencies of the variables ‘sex’, ‘age’ and ‘tumor type’.

Next, the data collected at the three time points were analyzed by grouping scores within the four assessed domains (Cog-PCI, Cog-O, Cog-PCA and Cog-Q). The mean and standard deviation were then calculated for each data category. The impact of the variable ‘sex’ on the mean scores was also examined for each dataset. The means obtained at the three time points were then compared to evaluate trends over time.

Subsequently, correlations between each independent variable (Cog-PCI, Cog-O and Cog-PCA) and the dependent variable (Cog-Q) were then investigated at a statistical significance level of 5% (α = 0.05). In this analysis, the effect of the ‘sex’ variable on the correlations was also examined.

Finally, a linear regression analysis was performed to assess the overall impact of all the independent variables on the dependent variable, considering a 95% confidence interval (CI) (α = 0.05). Correlations between individual variables and linear regression analyses were conducted separately for data collected at T0, T1 and T2.

## 4. Results

A total of 62 individuals were initially identified as potential participants in the study. Of these, 10 declined to participate, 7 did not complete one or more questionnaires due to discontinuation of therapy for toxicity affecting other organ systems, 3 continued therapy as inpatients in a different unit and 2 continued therapy at a different day hospital.

The final sample thus consisted of 40 participants. Baseline patient characteristics are reported in Table 1.

The sample included 29 females and 11 males, with a mean age of 50.65 years (range 26–64 years). Age distribution was as follows: 2% aged 25–34 years, 22% aged 35–44 years, 39% aged 45–54 years, and 37% aged 55–64 years. In terms of cancer type, 52% of the sample were women with breast cancer, 20% had lung cancer, 5% were women with ovarian or uterine cancer, 5% were men with testicular cancer and 5% had gastrointestinal tumors. The remaining 13% included participants with other types of cancer (e.g., leiomyosarcoma, osteosarcoma and liver cancer).

**Figure 1:**
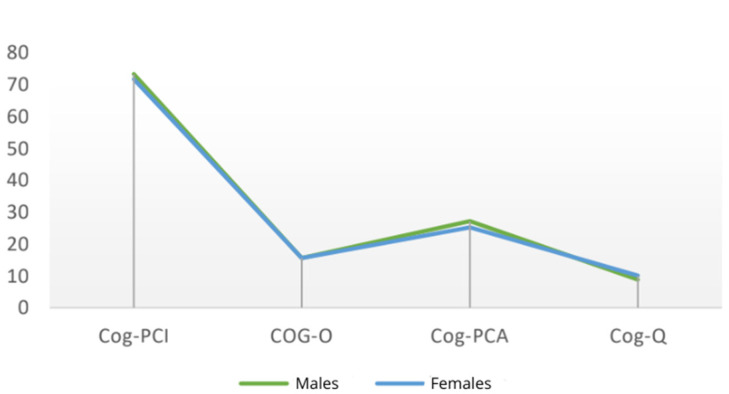
Mean scores of males and females at T0.

At baseline (T0), examining the impact of sex on the variables ‘Cog-PCI’, ‘Cog-O’, ‘Cog-PCA’ and ‘Cog-Q’, it was observed that men had higher mean scores in ‘Cog-PCI’ (73.27 ± 6.96) and Cog-PCA (27.18 ± 6.01), compared to women (Cog-PCI: 71.69 ± 9.79; Cog-PCA: 25.14 ± 8.27). However, women exhibited a higher mean score in Cog-Q (10.07 ± 5.20) than men (8.82 ± 5.44) (Figure 1).

Correlation coefficients were calculated between the independent variables ‘Cog-PCI’, ‘Cog-O’, and ‘Cog-PCA’ and the dependent variable ‘Cog-Q’, with a significance level of 5% (α = 0.05). None of the coefficients reached statistical significance even when accounting for the effect of the variable sex.

Linear regression analysis showed that at T0 ‘Cog-PCI’, ‘Cog-O’, and ‘Cog-PCA’ were not significant predictors of patients’ quality of life [F(4, 35) = 1.03, *p* = 0.4039]. The independent variables accounted for 10.5% of the variance in ‘Cog-Q’ (R^2^ = 0.1056). None of the predictors demonstrated a statistically significant effect on quality of life at the 5% significance level (α = 0.05) according to *t*-test statistics (Table 2).

**Figure 2:**
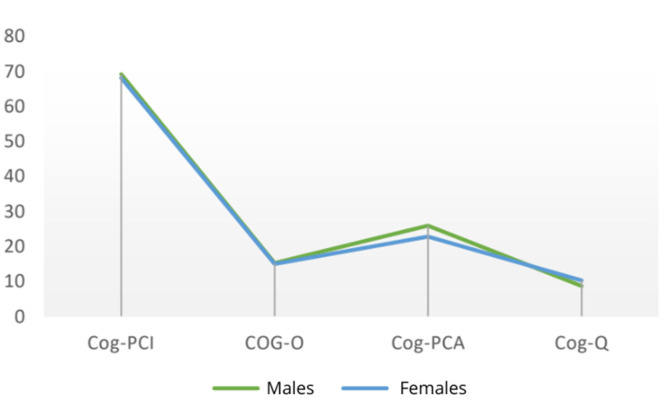
Mean scores of males and females at T1.

At the intermediate assessment (T1), analysis of the variable sex revealed that men once again demonstrated higher mean scores in Cog-PCI and Cog-PCA, whereas women reported a higher mean score for quality of life (Figure 2).

Individual correlation coefficients were calculated between the independent variables ‘Cog-PCI’, ‘Cog-O’ and ‘Cog-PCA’ and the dependent variable ‘Cog-Q’, considering a significance level of 5% (α = 0.05). At T1, the variable ‘Cog-PCA’ showed a statistically significant positive correlation with quality of life, both overall (coefficient = 0.3562) and when considering only the female population only (coefficient = 0.3942).

However, the linear regression analysis at T1 indicated that these variables did not predict patients’ quality of life with statistical significance [F(4, 35) = 2.33, *p* = 0.0755]. The independent variables accounted for 21.0% of the variance in ‘Cog-Q’ (R^2^ = 0.2101). Using the *t*-test statistic, it was found that variation in ‘Cog-PCA’ had a statistically significant impact on variation in quality of life (*p* > |t| = 0.018), considering a significance level of 5% (α = 0.05) (Table 3).

**Figure 3:**
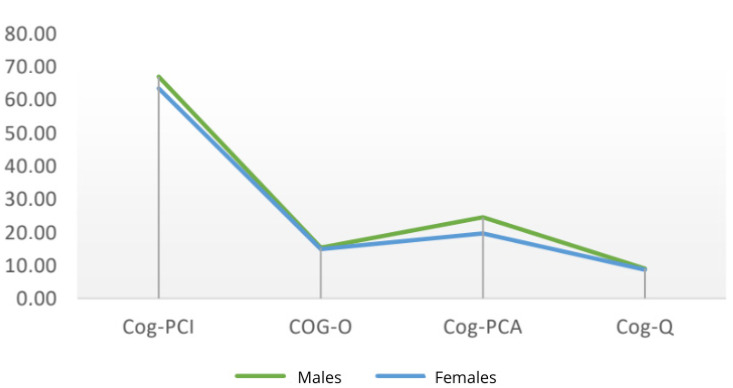
Mean scores of males and females at T2.

At the final assessment, men continued to have higher mean scores in “Cog-PCI” and “Cog-PCA,” while the previously observed difference in mean quality of life scores was minimal at T2 (Figure 3).

The correlation coefficients between the individual independent variables and the dependent variable ‘Cog-Q’ were recalculated at a significance level of 5% (α = 0.05). At T2, both ‘Cog-PCI’ (coefficient = 0.3652) and ‘Cog-PCA’ (coefficient = 0.5380) showed a statistically significant positive correlation with ‘Cog-Q’ in the entire sample. When stratified by sex, Cog-PCI correlated significantly with quality of life in men (coefficient = 0.6868), while Cog-PCA correlated significantly with quality of life in women (coefficient = 0.5573).

Linear regression analysis indicated that, at T2, the independent variables reliably predicted patients’ perceived quality of life (F(4, 35) = 3.91, *p* > F = 0.01), accounting for 30.9% of the variance in ‘Cog-Q’ scores (R^2^ = 0.3088). A *t*-test revealed that, at T2 Cog-PCA continued to have a statistically significant impact on ‘Cog-Q’ scores, i.e., on perceived quality of life (*p* > |t| = 0.005) (Table 4).

**Figure 4:**
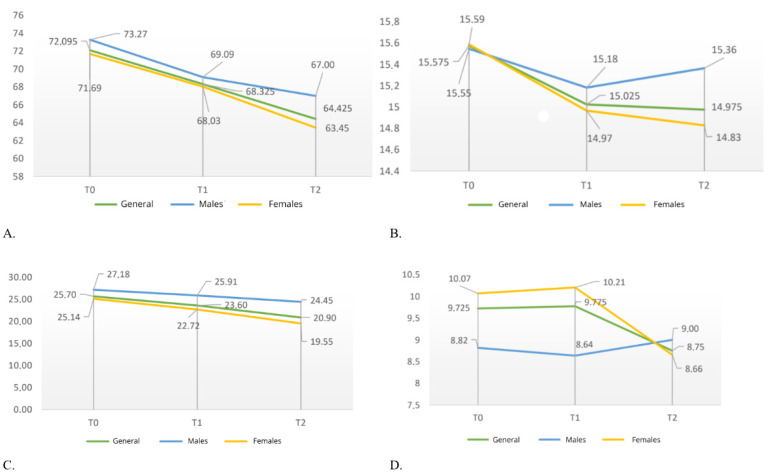
Change over time in the mean values of the overall sample, in males, and in females: (**A**). “Cog-PCI” variable; (**B**). “Cog-O” variable; (**C**). “Cog-PCA” variable; (**D**). “Cog-Q” variable.

When comparing the trend of mean ‘Cog-PCI’ scores over time, an overall decline of approximately 4 points per interval is observed. Among men, there is an initial 4-point decrease between T0 and T1, after which the decline in scores slows down. Among women, there is an initial mean decrease of 3 points between T0 and T1, followed by a further mean decline of approximately 5 points between T1 and T2 (Figure 4).

Regarding Cog-O scores, the overall decline from T0 to T2 was less than 1 point. In men, after an initial decline of 0.4 points at T1, the mean score at T2 is close to the baseline value. In women, however, despite initially having a similar mean score to that of the male population at T0, the mean scores progressively decrease at T1 and T2 (Figure 4).

Analyzing the trend over time in mean ‘Cog-PCA’ values shows that, in both the overall sample and the male and female subgroups, the mean score decreased linearly. However, males consistently achieved higher mean scores than females at all three time points (Figure 4).

Examination of mean quality of life scores over time reveals that females followed a trend similar to that of the overall sample, with a slight initial improvement between T0 and T1, followed by a marked decline at T2. By contrast, males showed a slight decline at T1, followed by an improvement at T2 (Figure 4).

## 5. Discussion

The findings further indicate that the mean FACT-Cog score progressively decreased during the first two months of chemotherapy, from 123.09 at T0 to 116.73 at T1 and 106.28 at T2 (Table 5). This suggests that patients’ perceived quality of life declines as chemotherapy progresses, which is probably due in part to the cognitive effects of antineoplastic drugs. These results are consistent with previous studies, although our sample differs in that much of the existing literature focuses on a single tumor type [25,26,27,28].

Interestingly, women consistently scored lower than men in the ‘Cog-PCI’ and ‘Cog-PCA’ domains at all three time points, yet reported higher average quality of life scores. This suggests that although women perceive a greater decline in their cognitive abilities, this has a smaller impact on their overall quality of life.

Consistent with findings in oncology research, women with cancer often report greater subjective cognitive difficulties than men, with studies showing that female patients paradoxically describe more pronounced cognitive complaints despite similar or even better objective performance, suggesting a sex-specific tendency for women to perceive and report cognitive changes more severely in the context of cancer and its treatment [29].

Our findings are in line with the broader oncology literature showing that women generally tend to report poorer self-perceived functioning and a higher symptom burden than men. This pattern supports the interpretation that female cancer patients may also perceive and report cognitive difficulties more severely, even when the overall impact on quality of life is attenuated or better compensated [30].

At T0, no correlation was observed between cognitive variables and quality of life, which is not surprising given that cognitive symptoms are likely to be mild or absent at diagnosis, while quality of life is significantly affected by stress and concern following a cancer diagnosis.

At both T1 and T2, however, a correlation emerged between ‘Cog-PCA’ scores, which reflect perceived cognitive abilities, and quality of life: the better the patient’s perceived cognitive abilities, the higher their reported quality of life. By T2, ‘Cog-PCI’ scores—reflecting perceived cognitive decline—also correlated with quality of life: fewer cognitive symptoms were associated with a better perceived quality of life.

Regression analyses at T1 and T2 further demonstrated that ‘Cog-PCA’ was the strongest predictor of quality of life, confirming its significant predictive power with regard to patients’ perceived quality of life (*p* > |t| = 0.005).

The association between ‘Cog-PCA’ and quality of life observed in this study has not been emphasized in previous research. However, this study differs from the existing literature in that assessments were conducted at shorter intervals over a more limited treatment period.

When considered individually, the other variables did not show a statistically significant impact on quality of life.

However, when the combined effect of perceived cognitive impairments, perceived cognitive abilities and comments from others was considered, these variables reliably predicted decline in quality of life at T2, accounting for approximately 30% of the variance in “Cog-Q”. These findings lead to two important conclusions. First, patients’ quality of life may be influenced by the perceived cognitive decline just two months after the start of antineoplastic therapy. Second, the FACT-Cog questionnaire appears to be a useful tool for rapidly assessing perceived cognitive decline in cancer patients, focusing specifically on the patient’s experience.

However, it is important to note that these factors account for less than one-third of the variance in ‘Cog-Q’, despite being good predictors. The literature suggests that multiple additional factors beyond cognitive decline contribute to patients’ quality of life [19,20,21,22,23,24,25,26,27,28,29,30,31,32].

These findings are consistent with the existing literature, emphasizing the negative impact of cognitive decline on quality of life. Moreover, the independent variables investigated (perceived cognitive impairments, comments from others and perceived cognitive abilities) reliably predicted changes in quality of life from the second month after therapy initiation. This suggests that it may be possible to identify patients at higher risk of reduced quality of life early on and intervene with targeted educational and rehabilitative strategies.

Furthermore, it emerged that perceived cognitive decline only partially explained the reduction in quality of life, suggesting that other factors—such as depression, stress, and anxiety—are probable contributors to adverse outcomes. Overall, these results underline the need for further research to better clarify the factors contributing to diminished quality of life in cancer patients.

## 6. Future Perspective: Early Cognitive Intervention Model

Patients experience cognitive impairments already within the first few months of chemotherapy; these deficits, which significantly affect quality of life, therefore require appropriate clinical management.

As mentioned, the Veneto Institute of Oncology has a neurocognitive clinic which uses standardized tools to assess cognitive disorders and implement cognitive rehabilitation programs, such as cognitive training and psychoeducational groups for patients and caregivers.

The study could be a starting point for identifying patients at risk among those undergoing chemotherapy treatment, using self-evaluation questionnaires.

This brief assessment allows for the early identification of potential risk indicators.

If a patient is found to be at risk, they can be referred to the neurocognitive outpatient clinic for a more comprehensive evaluation. This approach enables timely management of conditions that may progress, providing the opportunity to examine their longitudinal trajectories.

Following an in-depth evaluation involving neurocognitive testing, patients identified as high risk in this new model will be offered the opportunity to participate in a psychoeducational group. The primary aim of this intervention will be to enhance cognitive performance, perceived well-being, and quality of life (QoL), while also promoting adaptive changes in emotional and motivational attitudes. Supporting cognitive improvement in the early stages of chemotherapy is essential to prevent further impairment-related declines in QoL.

The psychoeducational intervention, which already exists for other patients, consists of eight structured biweekly 90 min sessions within a psychoeducational framework. Each session includes an initial theoretical section and a subsequent practical section. The theoretical component addresses metacognitive aspects, providing health education on cognitive functioning and coping strategies for everyday challenges. The practical component involves group discussions, during which participants have the opportunity to share and reflect on the difficulties they encounter in daily life.

The first session is focused on program orientation and participant introduction. The second session explores memory and personal experiences related to it, while the third and fourth sessions introduce and practice memory strategies. The fifth session addresses attention and concentration, emphasizing the cognitive skills underlying daily activities. The sixth session focuses on executive functions and everyday skills. The seventh session explores constructs of well-being, alongside exercises designed to enhance assertiveness and resilience. The eighth and final session involves post-assessment and group reflection to evaluate the effectiveness of the program.

In addition, the hospital psychology service provides care for patients who demonstrate a need for psychological support, focusing specifically on the experiences they undergo.

**Figure 5:**
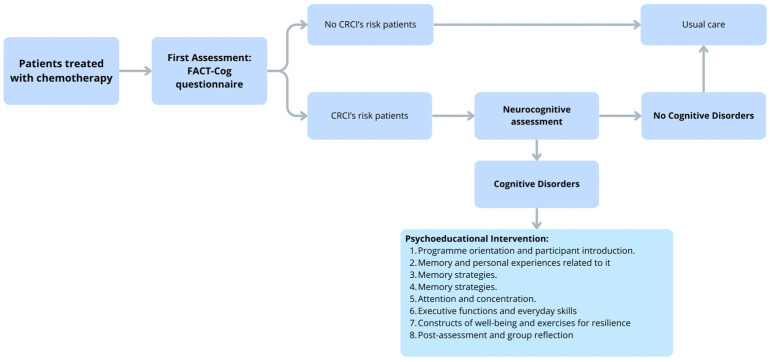
Chart of Preliminary Assessment and Early Intervention Model Flowchart.

Similarly, caregivers are offered the opportunity to engage in both psychoeducational and psychological programs from an early stage, as outlined in Figure 5.

## 7. Limitations

The main limitations of this study are the small sample size (40 participants), which may be subject to selection bias, and the short observation period of only two months, compared with the 6–24 months typically reported in similar studies. Nevertheless, this shorter time frame allowed us to investigate how cognitive decline and its impact on quality of life emerge in the earliest phases of treatment, and, as soon as this impairment was identified, early interventions were implemented to address it.

Its strengths lie in the inclusion of both men and women across different ages and cancer types—unlike most prior studies—and in its longitudinal design, which incorporated a baseline assessment to enable comparison across subsequent time points.

Nevertheless, an additional limitation is the unbalanced sex distribution of the sample, with a higher proportion of women than men; however, this distribution resulted from the consecutive and non-selective recruitment process and was not intentional. Future studies should aim to replicate these findings in larger samples with a more balanced sex distribution to further explore potential sex-related differences in perceived cognitive decline and quality of life.

Although the small sample size represents a limitation of the present study, it actually reflects the decision to provide these patients with early support from the outset, allowing for cognitive improvement aimed at mitigating the impact of cognitive decline on quality of life. Acting immediately is intended to prevent early cognitive decline, in combination with other previously mentioned factors, from reducing patients’ quality of life during the initial phases of treatment. This approach highlights the potential benefits of timely support for preserving patients’ cognitive functioning and well-being.

## 8. Conclusions

Over the past decade, chemotherapy-related cognitive decline has been the focus of numerous studies. It is estimated that 15–50% of cancer patients undergoing chemotherapy experience some form of cognitive impairment during treatment or in the following months. Such decline can substantially affect quality of life, making it difficult for patients to return to work, manage family responsibilities and maintain social relationships.

The present study examined cancer patients aged 18–64 receiving treatment at the Day Hospital of the Veneto Institute of Oncology, analyzing their perception of cognitive functioning during the first two months of chemotherapy. The study aimed to investigate how perceived cognitive abilities changed already in the early stages of chemotherapy treatment and over time with continued treatments and how these changes were associated with quality of life.

The data and subsequent statistical analyses highlighted two main findings. First, there was a general decline in both cognitive abilities and quality of life among chemotherapy patients. Second, there was a statistically significant correlation between perceived cognitive abilities and perceived quality of life: patients who perceived themselves as more competent in tasks requiring cognitive effort reported a higher quality of life.

The activation of early neurocognitive and psychoeducational interventions—such as the empowerment groups already implemented at the Veneto Institute of Oncology—appears essential to support patients experiencing cognitive difficulties. Such interventions, based on individualized assessment, may also help improve patients’ adaptation to treatment, promote psychological well-being, and enhance overall quality of life. The continuation of this line of research will make it possible to further evaluate the long-term benefits of early neurocognitive interventions and their integration into standard psychological care for cancer patients.

Ultimately, it becomes clear that comprehensive psychological care for cancer patients necessarily requires the evaluation and management of cognitive difficulties experienced throughout the course of the disease and its treatments. Addressing these aspects is a crucial step toward ensuring holistic and effective patient care.

## Figures and Tables

**Table 1:** Baseline characteristics of patients.

Variable	Category	N (%)
Number of patients	40	
Sex	Male	11 (28)
	Female	29 (72)
Age	Mean	50.65
	Range	65–84
	Range 25–34	1 (2)
	Range 35–44	9 (22)
	Range 45–54	16 (39)
	Range 55–64	14 (37)
Diagnosis	Breast cancer	21 (52)
	Lung Cancer	8 (20)
	Ovarian or uterine cancer	2 (5)
	Testicular cancer	2 (5)
	Gastrointestinal tract tumor	2 (5)
	Other types of cancer	5 (13)

**Table 2:** Coefficients of the independent variables and two-tailed T-statistic values at T0.

Variable	Coefficient	Standard Error	*p* > |t|
Sex	1.6459	1.8636	0.383
Cog-PCI	−0.0574	0.1502	0.704
Cog-O	0.3141	0.9557	0.744
Cog-PCA	0.2441	0.1601	0.136

**Table 3:** Coefficients of the independent variables and two-tailed T-statistic values at T1.

Variable	Coefficient	Standard Error	*p* > |t|
Sex	1.6459	1.8636	0.084
Cog-PCI	−0.0574	0.1502	0.22
Cog-O	0.3141	0.9557	−0.72
Cog-PCA	0.2441	0.1601	0.018

**Table 4:** Coefficients of the independent variables and two-tailed T-statistic values at T2.

Variable	Coefficient	Standard Error	*p* > |t|
Sex	1.6459	1.8636	0.084
Cog-PCI	−0.0574	0.1502	0.22
Cog-O	0.3141	0.9557	−0.72
Cog-PCA	0.2441	0.1601	0.018

**Table 5:** Mean and standard deviation of the variables at T0, T1 and T2.

	Time Point	Cog-PCI	Cog-O	Cog-PCA	Cog-Q	Total
Mean	T0	72.10 (±9.03)	15.08 (±1.01)	25.70 (±7.70)	9.73 (±5.23)	123.09 (±17.93)
	T1	68.33 (±9.18)	15.03 (±2.01)	23.60 (±9.03)	9.78 (±4.00)	116.73 (±16.05)
	T2	64.43 (±11.57)	14.98 (±1.51)	20.90 (±7.53)	8.75 (±4.11)	106.28 (±26.18)

## Data Availability

Due to privacy and ethical restrictions, the data that support the findings of this study are available on request from the corresponding author.

## Abbreviations

The following abbreviations are used in this manuscript:

CRCI	Cancer-Related Cognitive Impairments
FACT-Cog v.3	Functional Assessment of Cancer Therapy—Cognitive Function v.3
ICCTF	International Cognition and Cancer Task Force
